# Online Self-Regulated Learning Profiles: A Study of Chinese as a Foreign Language Learners

**DOI:** 10.3389/fpsyg.2021.797786

**Published:** 2021-12-13

**Authors:** Lin Lin, Yang Gong, Nuo Xu

**Affiliations:** ^1^International Cultural Exchange School, Shanghai University of Finance and Economics, Shanghai, China; ^2^Faculty of Education, University of Macau, Taipa, Macau SAR, China

**Keywords:** online self-regulated learning, motivated strategies for learning questionnaire, Chinese as a foreign language, latent profile analysis, motivation, learning strategies

## Abstract

The role of self-regulated learning (SRL) in achieving academic success has been widely investigated for campus-based college students. However, research on online learners’ SRL is limited, while the number of online learners has been increasing tremendously in recent years, especially after the COVID-19 pandemic worldwide. As SRL is context-bound, differences caused by classroom and home environments may be expected. This study investigated the factor structures of online learners’ SRL in Chinese as a foreign language education and the existence of SRL profiles in online learners. Data were collected from 378 international students enrolled in online Chinese language courses in 2020. Ten latent factors were revealed by exploratory factor analysis with motivation and learning strategies scales of the Motivated Strategies for Learning Questionnaire (MSLQ). A follow-up latent profile analysis showed three profiles of low, moderate, and high self-regulated learners. The study supports the context-bound nature of SRL and calls for developing adaptive training programs according to SRL profiles of Chinese language learners.

## Introduction

Over the past 20 years, the number of Chinese as a foreign language (CFL) or second language (CSL) learners has grown significantly within and outside China (Gong et al., 2020a,c, 2021c). In 2018, more than 500,000 international students participated in various courses and short-term immersion programs in over 820 educational institutions in mainland China (Ministry of Education, 2019). Influenced by the COVID-19 pandemic worldwide, international students enrolled in Chinese universities were required to learn Chinese language courses in their home countries via the internet (Ministry of Education, 2020). The nature of online classes is considerably different from face-to-face traditional classroom settings, which requires learners to be more autonomous and self-regulated (Stevens and Switzer, 2006).

Self-regulated learning (SRL) has received increasing attention in educational research due to its essential role in society today (Zimmerman, 2002; Heirweg et al., 2019). Effective SRL has been found to optimize learning processes and positively affect learning results in the traditional classroom (Dörrenbächer and Perels, 2016). Few studies have explored the SRL of non-traditional learners studying in an online learning environment. As SRL is context-bound, differences caused by classroom and home environment can be expected (Duncan and McKeachie, 2005; Meijs et al., 2019). Since studying online requires more SRL than in traditional education, it is necessary to determine whether the SRL structure developed for traditional college students is also suitable for online learners, especially for CFL/CSL learners (Gong et al., 2021a).

There is increasing evidence that individual differences exist in SRL (Barnard-Brak et al., 2010; Dörrenbächer and Perels, 2016). Learners may combine their motivation and learning strategies in a relatively unique way in SRL (Vansteenkiste et al., 2009). Most studies have explored SRL using a variable-centered approach instead of a person-centered approach. A person-centered approach should be adopted to identify different groups of online learners characterized by different SRL profiles.

Given the increasing demands of learning Chinese online since the outbreak of COVID-19, this study aimed to explore the SRL of international students in the context of learning CFL online in mainland China. The study also examined the SRL characteristics of different groups of online learners from a person-centered perspective. This contextualized examination offers new insights into the construct of SRL and produces practical implications for online Chinese language education.

## Literature Review

### Self-Regulated Learning

Self-regulated learning is “an active, constructive process whereby learners set goals for their learning and then attempt to monitor, regulate, and control their cognition, motivation, and behavior, guided and constrained by their goals and the contextual features in the environment” (Pintrich, 2000, p. 453). This conceptualization indicates that SRL is complex and multicomponent. Referring to Pintrich’s (2000) framework, SRL is composed of four stages: (1) forethought, planning, and activation; (2) monitoring; (3) control; and (4) reaction and reflection. Each stage has four different aspects for regulation: cognition, motivation/affect, behavior, and context. The combination of stages and aspects displays a comprehensive picture of a significant number of SRL processes (e.g., goal orientation adoption, monitoring of cognition, and self-observations of behaviors). The different SRL components for regulation are employed in the different stages.

A wildly used instrument to measure learners’ SRL is the Motivational Strategies for Learning Questionnaire (MSLQ) developed by Pintrich et al. (1991), established on the general cognitive model of motivation (Pintrich, 1988, 2003) and information processing (Weinstein and Mayer, 1986). This questionnaire has two subscales of motivation and learning strategies. Specifically, the motivation focuses on three constructs: (1) value, concerning the reason of learners’ engagement in a learning task, such as intrinsic and extrinsic goal orientation; (2) expectancy, referring to learners’ belief in accomplishing a task through their efforts or ability, such as self-efficacy; (3) affect, tapped into learners’ cognitive and emotional reactions to test anxiety. The subscale of learning strategies, built on the cognitive theory of learning (Bandura, 1986; Schunk, 2012), concerns three constructs: cognitive, metacognitive, and resource management strategies. Cognitive strategies include simple and complex strategies that learners use in a learning task, such as rehearsal and organization. Metacognitive strategies are applied by learners to regulate their cognitive behaviors, such as setting learning goals, monitoring learning processes, and modifying learning behaviors. Resource management strategies are non-cognitive strategies that learners utilize to regulate their learning, such as time, study environment, and peer learning. MSLQ is recognized as the most used instrument in SRL measurement (Roth et al., 2016). One of its major strengths is its combination of motivation and learning strategies, which provides learners with detailed information about their SRL.

Researchers have explored learners’ SRL behaviors across cultural contexts and population (Panadero, 2017; Tong et al., 2020). Many researchers have applied MSLQ to examine different groups of learners in many countries, such as Australia (e.g., Martin and Marsh, 2006), China (e.g., Tong et al., 2020), and Pakistan (e.g., Nausheen, 2016). Although there might be some universal constructs of SRL, researchers have found differences in factor structures and item assignments across culture and educational contexts (Nausheen, 2016; Ramírez Echeverry et al., 2016; Tong et al., 2020). Nausheen (2016) examined the factor structure of the motivation subscale of MSLQ through 368 graduate students in Pakistan. The factors of control of learning belief and intrinsic goal orientation were not found among this group of learners. Moreover, items of intrinsic goal orientation were loaded on the task value, indicating that the intrinsic motivation for the course was related to its overall utility and value. Ramírez Echeverry et al. (2016) collected the data from 1,218 engineering students in a Colombian university and found that the time and study environment in the learning-strategy scale was separated into two independent factors, i.e., time and study environment. In addition, the factors of peer learning and help-seeking were combined into one factor with all the original seven items, suggesting that learners considered these two resources similar.

In the context of mainland China, Tong et al. (2020) investigated 611 undergraduate students’ SRL behaviors in two coastal universities. The factor of intrinsic goal orientation was not identified, implying the interdependence between culture and SRL models. Peer learning and help-seeking were aggregated into one factor, indicating that Chinese adult learners did not differentiate between learning with peers and seeking help from peers. Time and study environment was split into two independent constructs, i.e., time management and study environment management, similar to those found in Ramírez Echeverry et al.’s (2016) study. Although time and study environment are two different resources learners should master ideally, the researchers argued that it was sufficient for learners to use either one properly to optimize their learning. Grounded in social cognitive theory, learners’ motivation and learning strategies in SRL are context-specific and influenced by culture (Zhou and Wang, 2021). Therefore, more research is needed to explore the constructs of MSLQ among different groups of learners in different educational contexts to better understand the transferability of SRL theory.

### Online Self-Regulated Learning

The COVID-19 outbreak worldwide has rapidly increased online learning, whereby digital technology facilitates teaching. Online learners choose the time, pace, and location to study and decide whether to contact teachers or peers of their wills (Eurydice, 2011). Distinct from the traditional face-to-face in-class education, online learning requires students to learn with more SRL strategies and self-motivation as they receive less support and guidance on how to learn efficiently (Zhou and Wang, 2021). As SRL is context-bound, different learning environments may lead to differences in motivation and learning strategy use between online and traditional learners (Duncan and McKeachie, 2005; Meijs et al., 2019).

Few empirical studies have explored the SRL behaviors of online learners. Since it is not practical to create a new instrument to measure existing SRL concepts, some researchers used the subscales of MSLQ to investigate online learning (e.g., Cho and Summers, 2012; Kizilcec et al., 2017). Cho and Summers (2012) used the original MSLQ to study learning strategies on online learning among 193 online learners at a large mid-western research university in the United States. The results indicated that the factor structure did not fit the sample well. As the MSLQ is initially designed to measure SRL among learners in traditional face-to-face education, some items in the questionnaire may not reflect the learning characteristics of online learners, especially their learning strategy use (Zhou and Wang, 2021). Recognizing this problem, Meijs et al. (2019) revised the learning strategy subscale of the MSLQ to apply to online education. They discovered that a 5-factor structure has a better fit than the original 9-factor model, namely, management of time and effort, simple cognitive strategy use, complex cognitive strategy use, contact with others, and academic thinking. Zhou and Wang (2021) validated Meijs et al.’s (2019) questionnaire using 385 students in an open university in China and found it had good validity and reliability. Five factors were generated from their data. They were named time management, effort regulation, cognitive strategy, critical thinking, and help-seeking, slightly different from the factors emerging from Meijs et al.’s (2019) study. Firstly, time management and effort management were split into two factors. Second, simple and complex cognitive strategies were aggregated into one factor. The researchers called for more empirical studies in different subjects to explore the SRL characteristics of online learners.

Most empirical studies have used a variable-centered approach to explore the different motivation and learning strategies that learners engage in separately (Cho and Summers, 2012; Meijs et al., 2019; Zhou and Wang, 2021). However, SRL learners may vary at the individual level. Even with the same learning context, students may have a different combination of motivation and learning strategies. A person-centered approach should be adopted to categorize individuals into groups with similar SRL profiles. Liu et al. (2014) investigated the SRL profiles of 238 college students using their MSLQ scores. They identified four subgroups: positive motivated strategies for learning, average motivated strategies for learning, low motivated strategies for learning with high anxiety, and negative motivated strategies for learning. Dörrenbächer and Perels (2016) collected the data from 337 college students and discovered four distinct SRL profiles: high SRL, conflicting SRL with high motivation, moderate SRL, and low SRL with moderate motivation. Few studies have adopted a person-centered approach to explore the SRL characteristics of online learners. Given that such diagnostic information may contribute to effective online instruction, additional research evidence is needed.

The COVID-19 pandemic outbreak forced many universities to remain closed temporarily in 2020 (UNESCO, 2020). In mainland China, almost half-million international students retreated to their countries and had to study online from their homes to continue their education (Bao, 2020). Since it is uncertain to get back to ordinary face-to-face teaching soon, online learning is promoted as a solution to teach international students in universities in China (Bao, 2020). As most international students deciding to study in Chinese universities involve Chinese language learning, it is necessary to investigate their online learning in Chinese language courses, especially for their SRL behaviors (Gong et al., 2021b). Moreover, as reviewed above, there lacks empirical research examining the SRL characteristics of online learners and their individual differences in SRL. Given these research gaps, this study is guided by the following two research questions:

RQ1: What are the characteristics of international students’ self-regulated learning in the context of online Chinese as a foreign language education?
RQ2: What are the distinctive profiles of international students concerning their self-regulated learning in the context of online Chinese as a foreign language education?

## Methodology

### Participants

A sample of 378 international students enrolled in higher-level education in mainland China participated in administering an online self-report SRL questionnaire. More particularly, 344 undergraduate and 34 graduate students took part, with a mean age of 22.49 years (*SD* = 4.24). There were 134 males (35.4%) and 234 females (64.6%). Most of the participants were from Teaching Chinese as a Foreign Language (TCFL) (*n* = 272). The rest were from different majors, such as Economics, International Business, and Law. On average, these participants had been learning Chinese in mainland China for 2.65 years (*SD* = 1.13) with 1,920 instructional hours at the time of the study. They all had passed Hanyu Shuiping Kaoshi (HSK) Level 4, a large-scale standardized Chinese proficiency test for non-Chinese learners in mainland China (Chinese Language Council International and Confucius Institute Headquarters, 2009). HSK Level 4 corresponds to Level B2 of the Common European Framework of Reference for Languages (CEFR) (Chinese Language Council International and Confucius Institute Headquarters, 2009). All the participants took at least one online Chinese language course in the autumn of 2020. Before that, they all have received at least 1 year of face-to-face classroom instruction in China. Appendix A displays the breakdown of the participants by country.

### Instrument

#### Motivated Strategies for Learning Questionnaire

The MSLQ (Pintrich et al., 1991) was used to measure the participants’ motivation and learning strategies in online Chinese language courses. The questionnaire contains two sections: motivation and learning strategies (Pintrich et al., 1991). The original 31 items in the motivation section were adopted in this study. These items assess students’ goal orientation and value beliefs for a course, the self-confidence of their ability to succeed in a course, and the anxiety of their academic performance. There are six factors in the motivation section: intrinsic goal orientation, extrinsic goal orientation, task value, control of learning beliefs, self-efficacy for learning and performance, and test anxiety. The learning strategies were measured with the adapted version of the MSLQ-B developed by Meijs et al. (2019), applied to online learners. This 25-item questionnaire consists of five factors: management of time and effort, complex cognitive strategy use, simple cognitive strategy use, contact with others, and academic thinking. Meijs et al. (2019) reported Cronbach’s alphas of factors ranging from 0.70 to 0.80. Strong validity and reliability support for this questionnaire were found in further empirical studies (Neroni et al., 2019; Zhou and Wang, 2021).

All questionnaire items were presented in both English and Chinese, with the English item corresponding to each Chinese item being provided to help participants accurately understand the items. The “translate and back translate” procedure was adopted to ensure a readable Chinese expression of the same meaning. Two bilingual scholars were invited to examine the accuracy of the translation. As the students could take several courses simultaneously in one semester, statements were stated generally instead of for a specific course. The participants rated themselves on a 7-point Likert scale, ranging from *not at all true of me* (1) to *very true of me* (7).

### Data Collection

A convenience sampling method was used to select the participants. The research team contacted Chinese language teachers in universities in mainland China through personal relationships and asked them to invite their students to complete the online questionnaire. Students were informed that their participation was completely voluntary and that their data would be kept confidential and used only for research purposes. Students filled out e-questionnaires through the link provided by the researchers and received e-learning materials as compensation for their participation. According to the information provided by the online questionnaire tool, most participants took approximately 20 min to complete the questionnaire.

### Data Analysis

Descriptive statistics of the questionnaire of items were first calculated, including means and standard deviations. The univariate and multivariate normality of the collected data were examined. Skewness and kurtosis were used to check for item-level univariate normality. Values of skewness between [–3, 3] and kurtosis between [–10, 10] were considered acceptable, indicating univariate normality (Kline, 2011). Mardia’s coefficient was used to examine multivariate normality. A value of 5.00 or below represented multivariate normality (Bentler, 2005). Confirmatory factor analysis (CFA) was then used to test whether the data in this study fit the original subscales of the motivation and learning strategies in the questionnaire (Pintrich et al., 1991; Meijs et al., 2019). However, the data failed to fit the pre-existing model. Thus, exploratory factor analysis (EFA) was performed to identify the factor constructs of motivation and learning strategies used in online language courses. CFA was later used to examine the latent factor structures hypothesized based on EFA results. EFA is an approach to explore the number of latent variables and possible underlying factor structures of a group of observed variables (Fabrigar et al., 1999). Principal axis factoring (PAF) was chosen as an extraction method as it is consistent with the common factor model (Fabrigar and Wegener, 2011). Oblimin rotation was selected because it allows factors to be correlated, as found in previous research (Neroni et al., 2019; Zhou and Wang, 2021). Items with factor loadings greater than 0.3 were considered meaningful. Correlations of factors were also calculated. CFA is a technique to examine the number of factors and the specification of factor loadings postulated by the researchers based on theoretical frameworks or/and empirical studies (Thompson, 2004). Maximum likelihood (ML) was chosen as the estimation method. Several goodness-of-fit indices (*x*^2^/*df* ≤ 3, Comparative Fit Index [CFI] ≥0.90, Goodness of Fit Index [GFI] ≥ 0.90, Root Mean Square Error of Approximation [RMSEA] ≥ 0.06, Standardized Root Mean Square Residual [SRMR] ≥ 0.08) were used to evaluate the fit of the hypothesized model (Hu and Bentler, 1999; Kline, 2011).

Latent profile analysis (LPA) was used to group students into homogenous classes with regard to SRL level. LPA is a probability-based approach to identify underlying group members showing similar patterns of continuous variables (Muthén, 2001; Magidson and Vermunt, 2004). The students’ responses to the factors generated from MSLQ were used to categorize them into groups that shared a similar degree of agreement on a particular combination of motivation and learning strategies. Maximum likelihood (ML) estimation, the most commonly used approach to estimate model parameters in LPA, was adopted to find the parameter estimates associated with the highest likelihood value coming from the sample (McLachlan and Peel, 2000; Pastor et al., 2007). Following the criteria set by Nylund et al. (2007), models from two to four profiles were tested to identify the number of profiles. The best-fitting model was decided by evaluating a combination of absolute (Vuong–Lo–Mendell–Rubin likelihood ratio test [VLMR-LRT] and Lo–Mendell–Rubin adjusted likelihood ratio test [LMRA-LRT]) and relative -(Akaike Information Criterion [AIC], Bayesian Information Criterion [BIC], Sample Size-adjusted Bayesian Information Criterion [SSA-BIC]) fit indices (Nylund et al., 2007). The non-significant *p-*value for VLMR-LRT and LMRA-LRT indicates that the estimated model with *k*-profiles fits the data better than the model with *k-1* profiles (Lo et al., 2001). Generally, lower AIC, BIC, ABIC values indicate better model fit, whereas higher entropy, usually closer to one, indicates high discrimination among the latent profiles (Muthén and Muthén, 2007).

To examine whether there were significant differences across profiles with distinct patterns in online SRL, a series of one-way analyses of variance (ANOVA) were performed. Profile membership serves as the independent variable and the identified underlying factors of motivation and learning strategies as the dependent variables. A Bonferroni correction (α = 0.05) was employed to find the statistical difference in the post-hoc test as well as to control for Type I error. Partial eta squared was used to measure effect sizes. A value lower than 0.06 was interpreted as a small effect, a value of 0.06–0.14 a medium effect, and a value higher than 0.14 a large effect when comparing the group differences (Cohen, 1988). LPA was conducted using Mplus 7 (Muthén and Muthén, 1998–2012), while all other data analyses were performed in SPSS 24 (International Business Machines (IBM), 2016).

## Results

### Factor Analyses

Before EFA, univariate normality was examined for the collected data. All items in the questionnaire were normally distributed, as the values for skewness and kurtosis were close to zero (Kline, 2011). Aligned with the two main scales of the MSLQ, the results are presented in two parts. First, EFA generated five factors in the analysis of the Motivation scale, accounting for 48.23% of the total variance. Six items were removed from the questionnaire due to their low factor loadings or cross-loadings on two factors. Referring to the original item assignments and interpretation (Pintrich et al., 1991), these five factors were labeled as extrinsic goal orientation, task value, control for learning beliefs, self-efficacy for learning and performance, and test anxiety. The original factor of intrinsic goal orientation did not emerge from the analysis. Two items of this factor (i.e., ‘The most satisfying thing for me in the course is trying to understand the content as thoroughly as possible,’ ‘When I have the opportunity in the class, I choose course assignments that I can learn from even if they don’t guarantee a good grade’) were found to load on the factor of task value. The other two items were deleted due to low factor loadings on any of the factors. The final motivation scale consists of five factors with 25 items. Mardia’s coefficient was 3.42, indicating the variables had multivariate normal distributions (Bentler, 2005). Later, the five-factor model was tested in CFA and found to have acceptable model fit (*x*^2^/*df* = 1.94, *p* < 0.001; CFI = 0.93; GFI = 0.91; RMSEA = 0.050; SRMR = 0.055). Table 1 presents the factor loading matrix of the five-factor solution.

**TABLE 1:** Exploratory factor analysis results for motivation items.

Factor	Item	Mean	*SD*	Factor loading					Reliability (α)
				1	2	3	4	5	
(1) Extrinsic Goal Orientation	(1) Getting a good grade in the class is the most satisfying thing for me right now.	5.46	1.37	0.56					0.72
	(2) The most important thing for me right now is improving my overall grade point average, so my main concern in the class is getting a good grade.	5.25	1.48	0.66					
	(3) If I can, I want to get better grades in the class than most of the other students.	5.33	1.43	0.61					
	(4) I want to do well in the class because it is important to show my ability to my family, friends, employer, or others.	4.97	1.63	0.55					
(2) Task Value	(5) If I study in appropriate ways, then I will be able to learn the materials in the course.	5.67	1.07		0.32				0.89
	(6) It is important for me to learn the course material in the class.	5.92	1.10		0.74				
	(7) I am very interested in the content area of the course.	5.55	1.24		0.71				
	(8) If I try hard enough, then I will understand the course material.	5.76	1.16		0.56				
	(9) I expect to do well in the class.	5.87	1.14		0.55				
	(10) The most satisfying thing for me in the course is trying to understand the content as thoroughly as possible.	5.65	1.17		0.59				
	(11) I think the course material in the class is useful for me to learn.	5.83	1.18		0.75				
	(12) When I have the opportunity in the class, I choose course assignments that I can learn from even if they don’t guarantee a good grade.	5.28	1.31		0.36				
	(13) I like the subject matter for the course.	5.61	1.16		0.79				
	(14) Understanding the subject matter of the course is very important to me.	5.81	1.13		0.82				
(3) Control for Learning Beliefs	(15) It is my own fault if I don’t learn the material in the course.	5.17	1.58			0.62			0.60
	(16) If I don’t understand the course material, it is because I didn’t try hard enough.	4.79	1.56			0.58			
(4) Self-Efficacy for Learning and Performance	(17) I believe I will receive an excellent grade in the class.	5.36	1.28				0.72		0.85
	(18) I’m certain I can understand the most difficult material presented in the readings for the course.	4.80	1.43				0.83		
	(19) I’m confident I can understand the most complex material presented by the instructor in the course.	4.84	1.33				0.79		
	(20) I’m confident I can do an excellent job on the assignments and tests in the course.	5.28	1.19				0.62		
	(21) I’m certain I can master the skills being taught in the class.	5.25	1.21				0.61		
(5) Test Anxiety	(22) When I take a test, I think about how poorly I am doing compared with other students.	3.79	1.78					0.35	0.72
	(23) When I take tests, I think of the consequences of failing.	4.34	1.80					0.57	
	(24) I have an uneasy, upset feeling when I take an exam.	4.29	1.69					0.84	
	(25) I feel my heart beating fast when I take an exam.	4.81	1.69					0.72	

In the analysis of the subscale of the learning strategies revised for online learners, EFA generated five factors, accounting for 43.01% of the total variance. Four items were deleted because their factor loadings were lower than 0.30. Based on the factor explanation of Meijs et al.’s (2019) questionnaire, these five factors were named as time management, effort regulation, Simple Cognitive Strategy Use, Contact with Others, and Academic Thinking. The original factor of management of time and effort was split into two factors in this study: time management and effort regulation. The original factor of complex cognitive strategy use was not identified. Three items were scattered among effort regulation (i.e., ‘When studying for this course, I try to determine which concepts I don’t understand well’), simple cognitive strategy use (i.e. ‘When reading for this class, I try to relate the material to what I already know’), and Academic Thinking (i.e., ‘I try to think through a topic and decide what I am supposed to learn from it rather than just reading it over when studying for this course’). The other two items were removed due to their low factor loadings. The final Learning Strategies scale includes five factors with 21 items. The value of Mardia’s coefficient was 4.28, indicating no violation of multivariate normality (Bentler, 2005). The goodness-of-fit indices of the CFA model were acceptable (*x*^2^/*df* = 2.76, *p* < 0.001; CFI = 0.92; GFI = 0.91; RMSEA = 0.058; SRMR = 0.066). Table 2 displays the factor loading estimates in the Learning Strategies scale. The correlations between factors generated from the MSLQ are shown in Table 3.

**TABLE 2:** Exploratory factor analysis results for items of learning strategies.

Factor	Item	Mean	*SD*	Factor loading					Reliability (α)
				1	2	3	4	5	
(1) Time Management	(1) I make good use of my study time for the course.	5.25	1.27	0.32					0.61
	(2) I rarely find time to review my notes or readings before an exam.	4.35	1.80	0.43					
(2) Effort Regulation	(3) I make sure that I keep up with the weekly readings and assignments for the course.	5.26	1.40		0.62				0.75
	(4) I attend this class regularly.	5.98	1.36		0.73				
	(5) Even when the course materials are dull and uninteresting, I manage to keep working until I finish.	5.54	1.11		0.61				
	(6) When studying for the course, I try to determine which concepts I don’t understand well.	5.20	1.25		0.51				
(3) Simple Cognitive Strategy Use	(7) When studying for the course, I read my class notes and the course readings over and over again.	5.10	1.29			0.54			0.79
	(8) When I study for the course, I go over my class notes and make an outline of important concepts.	5.07	1.29			0.83			
	(9) When reading for the class, I try to relate the material to what I already know.	5.28	1.21			0.45			
	(10) When I study for the course, I write brief summaries of the main ideas from the readings and my class notes.	4.84	1.39			0.55			
	(11) Whenever I read or hear an assertion or conclusion in the class, I think about possible alternatives.	4.85	1.22			0.32			
	(12) I make lists of important item for the course and memorize the lists.	4.84	1.38			0.73			
(4) Contact with Others	(13) I try to work with other students from the class to complete the course assignments.	4.14	1.68				0.88		0.67
	(14) When studying for the course, I often set aside time to discuss course material with a group of students from the class.	3.88	1.64				0.50		
	(15) I try to identify students in the class whom I can ask for help if necessary.	4.64	1.60				0.45		
(5) Academic Thinking	(16) When I study the readings for the course, I outline the material to help me organize my thoughts.	4.85	1.41					0.44	0.74
	(17) When reading for the course, I make up questions to help focus my reading.	4.74	1.35					0.44	
	(18) I often find myself questioning things I hear or read in the course to decide if I find them convincing.	4.37	1.04					0.58	
	(19) When a theory, interpretation, or conclusion is presented in class or in the readings, I try to decide if there is good supporting evidence.	4.79	1.14					0.47	
	(20) I treat the course material as a starting point and try to develop my own ideas about it.	4.88	1.33					0.48	
	(21) I try to think through a topic and decide what I am supposed to learn from it rather than just reading it over when studying for the course.	4.75	1.30					0.71	

**TABLE 3:** Correlation matrix of the SRL factors.

	EGO	TV	CLB	SLP	TA	TM	ER	SCSU	CO	AT
EGO	1.00									
TV	0.46**	1.00								
CLB	0.20**	0.45**	1.00							
SLP	0.38**	0.60**	0.34**	1.00						
TA	0.30**	0.07	0.11*	–0.07	1.00					
TM	0.10	0.41**	0.16**	0.24**	−0.13*	1.00				
ER	0.31**	0.57**	0.25**	0.43**	0.10*	0.39**	1.00			
SCSU	0.37**	0.56**	0.25**	0.42**	0.15**	0.28**	0.59**	1.00		
CO	0.29**	0.17**	0.08	.17**	0.17**	–0.06	0.16**	0.34**	1.00	
AT	0.37**	0.51**	0.23**	0.35**	0.10	0.20**	0.40**	0.64**	0.43**	1.00

*EGO, extrinsic goal orientation; TV, task value; CLB, control for learning beliefs; SLP, self-efficacy for learning and performance; TA, test anxiety; TM, time management; ER, effort regulation; SCSU, simple cognitive strategy use; CO, contact with others; AT, academic thinking.*

*p < 0.05, **p < 0.01.

### Latent Profile Analysis

Based on the results of CFA, LPA was conducted with SRL factors as indicator variables. A three-profile model showed a robust statistical fit to the data. The entropy value for a three-profile model was high (0.847), indicating the precision of assigning individuals to their respective groups. The values of AIC, BIC, and SSA-BIC decreased considerably at the three-profile model. The results of VLMR-LRT and LMRA-LRT showed that the three-profile model had a better fit than the two- and four-profile models. The fit indices for the three latent profile models are shown in Table 4.

**TABLE 4:** Fit statistics for latent profile analysis.

Model	Profile size (*n*)	Free parameters	Entropy	AIC	BIC	SSA-BIC	VLMR-LRT (p)	LMRA-LRT (*p*)
2-profile	182/196	31	0.812	10478.12	10600.09	10501.74	<0.01	<0.01
3-profile	93/120/165	42	0.841	10280.45	10445.72	10312.47	<0.01	<0.01
4-profile	49/65/128/136	53	0.817	10198.88	10407.43	10239.27	0.493	0.499

*AIC, Akaike’s Information Criterion; BIC, Bayesian Information Criterion; SSA–BIC, sample size adjusted Bayesian Information Criteria; VLMR-LRT(p), p-values for the Vuong-Lo–Mendell–Rubin likelihood ratio test for K versus K- 1 profiles; LMRA-LRT (p), p-values Lo–Mendell–Rubin adjusted likelihood ratio test for K versus K-1 profiles.*

Table 5 presents means and standard deviations of SRL factors for the three profile groups. The first group showed the lowest values on all ten factors, which was named as the low SRL group (*n* = 120, 31.7%). The second group had moderate values on most subscales with the lowest value on the factor of contact with others. Thus, this group was named as moderate SRL group (*n* = 165, 43.7%). The third group was described as high SRL (*n* = 93, 24.6%) as this group had high values on all subscales except the factor of Test Anxiety. A series of ANOVAs were conducted to examine if there were significant associations between profile membership and ten SRL factors. Significant differences were revealed between the groups on the factors of extrinsic goal orientation [*F*(2,375) = 65.20, *p* < 0.001, η^2^ = 0.25], task value [*F*(2,375) = 294.74, *p* < 0.001, η^2^ = 0.61], control for learning beliefs [*F*(2,375) = 43.56, *p* < 0.001, η^2^ = 0.19], self-efficacy for learning and performance [*F*(2,375) = 110.91, *p* < 0.001, η^2^ = 0.37], time management [*F*(2,375) = 31.08, *p* < 0.001, η^2^ = 0.14], effort regulation [*F*(2,375) = 213.84, *p* < 0.001, η^2^ = 0.53], simple cognitive strategy use [*F*(2,375) = 291.93, *p* < 0.001, η^2^ = 0.61], Contact with Others [*F*(2,375) = 34.80, *p* < 0.001, η^2^ = 0.16], and Academic Thinking [*F*(2,376) = 147.29, *p* < 0.001, η^2^ = 0.44]. There was no statistically significant difference on the factor of test anxiety, as students in all three groups showed relatively low degree of anxiety.

**TABLE 5:** Factor mean scores across three latent profiles.

Factor	Profile					
	Low SRL (*n* = 120)		Moderate SRL (*n* = 165)		High SRL (*n* = 93)	
	Mean	*SD*	Mean	*SD*	Mean	*SD*
(1) Extrinsic Goal Orientation	4.65	0.88	5.21	1.07	6.10	0.78
(2) Task Value	4.80	0.66	5.90	0.47	6.47	0.37
(3) Control for Learning Beliefs	4.19	1.23	5.15	1.21	5.68	1.12
(4) Self-Efficacy for Learning and Performance	4.27	0.78	5.24	0.85	5.92	0.79
(5) Test Anxiety	4.09	1.01	4.37	1.35	4.47	1.47
(6) Time Management	4.20	0.90	4.91	1.18	5.39	1.24
(7) Effort Regulation	4.52	0.73	5.73	0.68	6.32	0.52
(8) Simple Cognitive Strategy Use	4.19	0.53	4.95	0.65	6.13	0.51
(9) Contact with Others	3.94	0.95	3.92	1.28	5.10	1.22
(10) Academic Thinking	4.17	0.59	4.62	0.67	5.64	0.61

## Discussion

This study investigated the SRL characteristics of international learners in online CFL courses. A person-centered approach was adopted to examine how homogenous subgroups of individuals combine several SRL strategies differently. The findings highlight several differences and similarities concerning those documented in previous studies.

### Self-Regulated Learning Constructs of Online Learners

In terms of the first research question, the EFA analysis generated five factors for the motivation subscale: extrinsic goal orientation, task value, control for learning beliefs, self-efficacy for learning and performance, and test anxiety. The original factor of intrinsic goal orientation was not identified in this study. Two original items of this factor (i.e., ‘The most satisfying thing for me in the course is trying to understand the content as thoroughly as possible,’ ‘When I have the opportunity in the class, I choose course assignments that I can learn from even if they do not guarantee a good grade’) were found to load on the factor of the task value. The result is consistent with the findings of previous studies conducted with on-campus students in different contexts, such as Pakistan (Nausheen, 2016) and China (Tong et al., 2020). These two items focused on the learners’ evaluation of how interesting and valuable the online course was. Such statements did not strongly indicate that the learners participated in the online course for the challenge, curiosity, and mastery. They failed to distinguish between intrinsic goal orientation and task value that refer to learners’ perception of the course regarding its interest and usefulness (Tong et al., 2020).

Self-regulated learning theories and cultural differences may explain the absence of intrinsic goal orientation found in this study. Theoretically, context or external evaluation also plays an essential role in developing and adapting learners’ SRL competence (Efklides, 2011). Moreover, the extrinsic motivation that drives learners to study to achieve a goal or participate in a task has been found to promote their learning (Eisenberger and Cameron, 1996; Greene et al., 2004). From the perspective of culture, two-thirds of the participants are from Asia. They may grow up in societies pursuing goals set or approved by families (Yu and Yang, 1994). They are likely to choose to learn Chinese language courses to fulfill their parents’ expectations (Tang and Neber, 2008). Participation in learning online Chinese courses is also compelled by social reasons, such as receiving good grades, pleasing others, gaining approval, or earning social status, all of which are typical representations of extrinsic motivation (Wang and Lu, 2016). Social and familial influences may make Asian learners more external-goal-oriented (Tong et al., 2020). The lack of intrinsic goal orientation is also aligned with empirical results in cross-cultural validation between Chinese (Tong et al., 2020) and Pakistani (Nausheen, 2016) college students. More research is recommended to explore the constructs of online SRL with various populations.

The EFA analysis generated five factors for the learning strategies scale: time management, effort regulation, simple cognitive strategy use, contact with others, and academic thinking. The first difference is the split of time and effort management into two factors: time management and effort regulation. This result is similar to those studies reported among Spanish and Chinese learners (Ramírez Echeverry et al., 2016; Tong et al., 2020; Zhou and Wang, 2021). The online learners in this study consider the management of time and effort as two different resources. Ideally, learners should manage both resources properly. However, they may only use one of them appropriately. Online learners have the autonomy to manage learning time flexibly devoted to course learning (Kenner and Weinerman, 2011) or regulate their effort adaptably to learn better (Panadero, 2017). Notably, effort regulation is recognized as one of the most crucial SRL strategies for online learners (Kizilcec et al., 2017). In online learning, learners’ persistence against distractions or obstacles when watching videos or working on tedious tasks leads to their success (Lee et al., 2019). Therefore, it is reasonable to separate factors. The result also supports the SRL theory of learners’ effort to improve their learning (Panadero, 2017).

The second difference from the original scale is the absence of complex cognitive strategy use in this study. Items were scattered among effort regulation, simple cognitive strategy use, and academic thinking. The original factor of complex cognitive strategy use was not identified. Some items were loaded on other factors. The result supports Zhou and Wang’s (2021) argument that cognitive strategy use is complex. It is challenging to distinguish complex cognitive strategies from other learning strategies, especially in relatively large samples in the online context (Zhou and Wang, 2021). For example, one original item (i.e., ‘I try to think through a topic and decide what I am supposed to learn from it rather than just reading it over when studying for this course’) was loaded on the factor of academic thinking. This item focuses on critically evaluating the course material and using it as a beginning for intertwining the information with previous and common knowledge (Meijs et al., 2019). Such a statement does not seem to be a strong indicator of organizing or elaborating information. Therefore, it is reasonable to assign this item to academic thinking. The unidentified complex cognitive strategy use may also suggest that its components are separate and not based on a similar latent construct among international students in online Chinese language courses. The different factors of SRL that emerged in the study underline the importance of exploring the internal structure of SRL across cultural contexts and population.

### Latent Profile Analysis

Latent profile analysis was conducted to address the second research question. The results indicated variability in the SRL of online learners within the same learning context. Three distinct profiles of online learners were found, namely, the *low*, *moderate*, and *high* SRL groups. Online learners who were assigned to the *high SRL* profile, which was the smallest profile group, showed higher scores for both the motivation and the learning strategies. The *post hoc* tests indicated that these online learners had significantly higher scores in all factors than those in the *low* and *moderate* profiles, except for Test Anxiety. Individuals in this profile had the high motivation and regulated their online learning strategically. Nearly half of the online learners were described as *moderate SRL*. Online learners in this group gave relatively average scores to the factors of motivation and learning strategies except for test anxiety and contact with others. They reported the lowest score on contact with others among the three profiles, implying they preferred studying independently to making contact with others. Over 30% of the participants in the sample were categorized into the *low SRL* profile. This finding indicated that quite some online learners were less motivated and applied learning strategies less frequently and effectively.

It should be noted that online learners across the three profiles gave relatively low ratings to items under the factor of test anxiety. It is reasonable that online learners in the *high SRL* profile have low test anxiety as they are highly motivated and capable of regulating their online learning process. The low anxiety toward assessment performance among the *low* and *moderate SRL* learners can be signs of apathy (Liu et al., 2014). Another plausible explanation is the differences in the test-delivery format. Online learners can take the tests almost anywhere they have electronic devices and internet connection. Thus, they can choose an environment less likely to evoke their anxiety experienced in the traditional classroom in the past (Stowell and Bennett, 2010). Taking the test online may also decrease the memory retrieval signals available to the online learners and thereby balance any performance improvement based on the context-dependent memory influence (Godden and Baddeley, 1975). Allowing students to control their test-taking environment may help learners reduce their test anxiety (Lazarus, 1999; Yang and Taylor, 2013). Apart from these possible explanations, it is possible that test anxiety in the online environment is associated with other variables, for example, students’ perceptions of course difficulty in the online context (Neroni et al., 2019). Other influencing factors should be potential subjects of future research to explain the low test anxiety found among online learners.

Notably, compared with those in the *high SRL* group, learners in the *low* and *moderate SRL* groups rated significantly lower on contact with others. Unlike traditional face-to-face classroom learning, where students meet each other daily, online learning requires them to undertake extra actions to reach out to their peers or teachers (Meijs et al., 2019). Learners may be discouraged from seeking help in an online course because physical proximity to their teachers and classmates is limited (Yang and Taylor, 2013). Lane and Henson (2012) found that students in online classes reported less attachment to their classmates and university than did students who study in the traditional classroom. Especially for struggling learners, like those in the *low* and *moderate* SRL groups with low self-efficacy, they may be less likely to contact others for help when they do not believe that making the extra effort would lead to better performance (Roussel et al., 2011). The results are consistent with previous findings that low self-efficacy results in avoiding help-seeking, further inhibiting success (Roussel et al., 2011; Yang and Taylor, 2013).

The SRL profiles yielded in LPA underline the urgent need to promote SRL in online language courses as over 30% of the students in this study are categorized into the *low SRL* profile. The 30% share of low SRL learners may still underestimate the actual situation, as previous studies indicate that students were likely to overestimate their SRL behaviors in self-report questionnaires (Boekaerts and Corno, 2005; Heirweg et al., 2019). Prior research shows that SRL can be promoted in the traditional classroom context by implementing instructional skills (Perry et al., 2004; Dignath-van Ewijk et al., 2013; Dörrenbächer and Perels, 2016; Heirweg et al., 2019). For example, teachers may support students’ SRL by using a cognitive strategy while verbalizing or describing the usefulness of a learning strategy while encouraging students to apply it (Perry et al., 2004; Heirweg et al., 2019). Since online learning is more complicated than traditional classroom learning, in which students receive less support and guidance from their teachers and peers, more intervention-based research is needed to explore how teachers can effectively implement their instruction to promote SRL in online Chinese language courses.

## Conclusion

This study explored the SRL characteristics of international students in online CFL language courses in mainland China and further investigated the variability of these SRL characteristics using a person-centered perspective. The results showed five distinct factors for the scales of motivation and the learning strategies, respectively, supporting the context-dependent nature of SRL. Three groups of online learners were identified, indicating that not all online learners owned similar SRL characteristics, even from the same learning context.

Despite the significance of the findings in this study, two aspects may be optimized in future studies. First, the data are only collected through the self-reported SRL questionnaire. As the self-reported questionnaire usually depends on learners’ general understanding of their behaviors, it tends to be memory distortions (Veenman, 2011). Future studies are recommended to combine the data collected from the self-reported questionnaire with other more objective data, such as interviews, peer ratings, or observations. Gaining more objective data allows for cross-validation of the findings of online learners’ SRL behaviors in Chinese language courses. Second, two factors only have two items loaded and one factor has three items, which may explain the low reliability of the respective factor. The limited number of indicators may be caused by removing items with low factor loadings (i.e., control of learning beliefs) and separating one factor into two (i.e., management of time and effort into factors of time management and effort regulation). Although these factors had high theoretical relevance themselves and showed relatively low and non-significant correlation with other factors, their reliability coefficients did not reach 0.70 and therefore, should be cautiously interpreted (Taber, 2018). Future studies are recommended to add more items to enrich these factors and further validate MSLQ among online learners. Third, online learners’ general SRL characteristics in Chinese language courses are explored instead of specific courses. Different courses, focusing on different language skills, such as speaking, listening, or reading, may result in learners’ various SRL behaviors (Duncan and McKeachie, 2005). It is better to measure online learners’ SRL characteristics at a course level instead of a general level. At the same time, longitudinal research is needed to examine the influence of online SRL experiences on the participants’ motivational sustainability and their language proficiency development (Gong et al., 2020d).

Despite these limitations, there are several implications for future research and Chinese language education. First, the motivation and learning strategies that emerged from online learners in this study contribute to understanding SRL in different contexts. Second, SRL training programs need to be tailored to different learners for online Chinese language education. Language teachers are recommended to pay attention to each group of learners’ specific needs and design the programs to foster their SRL more effectively (Gong et al., 2020b, 2021b,c). Last, the accessibility and understanding of SRL theories can be advanced using a person-centered approach.

With the popularization and application of online language education in China, it is of great significance to understand how learners self-regulate their motivation and behaviors in the online learning context. This study provides a preliminary result concerning international students’ SRL in the online Chinese language courses. Future research could investigate this group of learners’ SRL alongside their academic performance to better understand the relationship between SRL and language achievement. Future studies may also compare the SRL characteristics of online learners and traditional learners in learning Chinese to explore possible models using MSLQ inventory across different learner groups.

## Data Availability Statement

The raw data supporting the conclusions of this article will be made available by the authors, without undue reservation.

## Ethics Statement

The studies involving human participants were reviewed and approved by Faculty of Education, The University of Hong Kong. The patients/participants provided their written informed consent to participate in this study.

## Author Contributions

LL conceived and designed the analysis, performed the data, and wrote the manuscript. YG revised the manuscript. NX collected the data and designed the analysis. All authors contributed to the article and approved the submitted version.

## Conflict of Interest

The authors declare that the research was conducted in the absence of any commercial or financial relationships that could be construed as a potential conflict of interest.

## Publisher’s Note

All claims expressed in this article are solely those of the authors and do not necessarily represent those of their affiliated organizations, or those of the publisher, the editors and the reviewers. Any product that may be evaluated in this article, or claim that may be made by its manufacturer, is not guaranteed or endorsed by the publisher.

## Appendix

**Appendix A:** Participants by country.

Country	Frequency	Percent
Antiguan	1	0.3
Argentina	4	1.1
Armenia	3	0.8
Azerbaijan	1	0.3
Bangladesh	5	1.3
Belgium	1	0.3
Brazil	2	0.5
Burma	3	0.8
Cambodia	5	1.3
Chile	1	0.3
Cyprus	1	0.3
Ecuador	3	0.8
Egypt	9	2.4
El Salvador	1	0.3
Estonia	3	0.8
Fiji	1	0.3
France	2	0.5
Georgia	1	0.3
Guinea	1	0.3
Hungary	2	0.5
Indonesia	23	6.1
Italy	2	0.5
Japan	17	4.5
Kazakhstan	10	2.7
Korea	30	7.9
Kyrgyzstan	9	2.4
Laos	12	3.2
Madagascar	1	0.3
Malaysia	24	6.3
Mauritania	1	0.3
Mauritian	1	0.3
Mexico	3	0.8
Mongolia	5	1.3
Morocco	1	0.3
Mozambique	2	0.5
Myanmar	1	0.3
Nepal	5	1.3
Netherland	1	0.3
New Zealand	1	0.3
Nigeria	2	0.5
Pakistan	5	1.3
Peru	5	1.3
Philippines	9	2.4
Poland	1	0.3
Portugal	1	0.3
Romania	1	0.3
Russia	27	7.1
Rwanda	1	0.3
Singapore	1	0.3
Spain	2	0.5
Sri Lanka	2	0.5
Sudan	2	0.5
Syria	2	0.5
Tajikistan	8	2.1
Tanzania	1	0.3
Thailand	57	15.1
Turkey	3	0.8
Turkmenistan	2	0.5
Ugandan	1	0.3
Ukraine	12	3.2
United Kingdom	4	1.1
United States	4	1.1
Uzbekistan	8	2.1
Vietnam	17	4.5
Yemen	1	0.3

**Total**	**378**	**100.0**
